# The Beneficial Effects of Ultramicronized Palmitoylethanolamide in the Management of Neuropathic Pain and Associated Mood Disorders Induced by Paclitaxel in Mice

**DOI:** 10.3390/biom12081155

**Published:** 2022-08-20

**Authors:** Claudia Cristiano, Carmen Avagliano, Mariarosaria Cuozzo, Fabrizio Maria Liguori, Antonio Calignano, Roberto Russo

**Affiliations:** Department of Pharmacy, University of Naples Federico II, Via D. Montesano 49, 80131 Naples, Italy

**Keywords:** paclitaxel, um-PEA, inflammation, neuropathic pain, behavior, cannabinoids, PPAR-α

## Abstract

Chemotherapy-induced peripheral neuropathy (CIPN) is a common complication of antineoplastic drugs, particularly paclitaxel (PTX). It can affect the quality of patients’ lives and increase the risk of developing mood disorders. Although several drugs are recommended, they yielded inconclusive results in clinical trials. The aim of the present work is to investigate whether the palmitoylethanolamide (PEA) would reduce PTX-induced CIPN and associated mood disorders. Moreover, the role PPAR-α and the endocannabinoid system will also be investigated. CIPN was induced by intraperitoneally injection of PTX (8 mg/kg) every other day for a week. PEA, 30 mg/kg, was orally administrated in a bioavailable form (i.e., ultramicronized PEA, um-PEA) one hour after the last PTX injection, for 7 days. In the antagonism experiments, AM281 (1 mg/kg) and GW6471 (2 mg/kg) were administrated 30 min before um-PEA. Our results demonstrated that um-PEA reduced the development of hypersensitivity with the effect being associated with the reduction in spinal and hippocampal pro-inflammatory cytokines, as well as antidepressive and anxiolytic effects. Moreover, the PPAR-α and CB1 receptor antagonists blocked the behavioral and antinociceptive effects of um-PEA. Our findings suggest that um-PEA is a promising adjunct in CIPN and associated mood disorders through the activation of PPAR-α, which influences the endocannabinoid system.

## 1. Introduction 

Cancer is one of the leading causes of death worldwide. In the last few years, the survival rates for most cancers have been increasing, probably due, at least in part, to new and improved treatments [1]. Despite the large interest in new drugs to fight cancer, old drugs still have an important use due to their high efficacy, despite their marked toxicity.

Paclitaxel (PTX) is a taxane chemotherapeutic agent used in the treatment of several cancer types. Although it shows beneficial antitumoral effects, it also produces important secondary effects both in the central and peripheral nervous system, resulting in emotional deficits and peripheral neuropathy, respectively [1,2,3]. Specifically, PTX causes chemotherapy-induced neuropathic pain (CIPN), a condition characterized by thermal and mechanical allodynia and hyperalgesia, which often persists for a long time [4,5]. In this case, the severity of symptoms can greatly increase the risk of developing mood disorders, including anxiety and depression, reinforcing the conception of CIPN as a chronic disease [6,7,8]. 

Unfortunately, there are currently no clinically effective interventions for CIPN, the efficacy of the existing therapies being only moderate [9]. 

PTX-induced neuropathy is initially characterized by oxidative stress followed by mitochondrial dysfunction [1,10,11,12,13]. Moreover, chemotherapeutical agents such as PTX have been shown to act similarly to LPS, i.e., increasing the production and release of pro-inflammatory interleukin (IL)-6 and IL-8 [14,15]. Notably, PTX-induced inflammation often interferes with its typical clinical efficacy, increasing tumor proliferation or chemoresistance [16,17,18] and contributing to the development of toxicity. Indeed, inflammation and neuroinflammation are a prominent characteristic of pain [19], as well as mood disorders [20]. Although acute inflammation can be considered a protective mechanism, chronic inflammation creates an array of detrimental effects. Despite the increasing understating of these mechanisms, novel analgesic strategies for treating PTX-induced toxicity are still lacking, and very few studies have investigated new possible effective treatments for CIPN and associated emotional components.

Palmitoylethanolamide (PEA) is a bioactive lipid mediator [21,22] belonging to the *N*-acyl-ethanolamine (NAE) family [23]. Although it is recognized that PEA primarily targets the nuclear receptor peroxisome proliferator-activated receptor alpha (PPAR-α), other receptors have been shown to mediate, at least in part, the effects of PEA, including G protein-coupled receptor 55 (GPR55), G protein-coupled receptor 119 (GPR119), and the transient receptor potential vanilloid receptor 1 (TRPV1) channels. Additionally, PEA indirectly activates cannabinoid receptors through the increase in the level of endocannabinoid mediators, such as anandamide (AEA), and 2-arachidonoylglycerol (2-AG) through the so-called called ‘entourage effect’ [24]. The well-known anti-inflammatory and antinociceptive effects exerted by PEA are considered to be an important non-pharmacological strategy in the management of neuropathic pain conditions, shown by several studies performed in preclinical models of inflammatory [25] and neuropathic pain [26,27,28,29,30,31], as well as clinical studies on human patients affected by osteoarthritis [32,33], neuropathic pain [34,35,36], fibromyalgia [37], and endometriosis [38]. Importantly, pain insensitivity in a human patient has been recently shown to be associated to a genetic deficiency in PEA-degrading enzymes, resulting in significantly higher PEA plasma levels compared to age-matched control patients [39].

In fact, PEA efficacy in chemotherapy-induced neuropathy has already been evaluated by previous studies via the oxaliplatin- [40] and PTX-induced CIPN model [41]. In particular, the last study has shown the effect to be mediated by PPAR-α, whose antagonism (i.e., through fenofibrate) is indeed known to reduce neuroinflammation in PTX-induced neuropathy [42]. 

PEA and its receptors are present in the central nervous system (CNS) [43,44], and exogenously administrated PEA is known to cross the blood–brain barrier [45], thereby exerting neuroprotective actions and promoting the resolution of neuroinflammation [46], and the regulation of behavior, mood, and cognition [47]. The therapeutic use of PEA in CNS disorders has produced promising results in several conditions, ranging from depression [48] to post-traumatic stress disorder [49] and autism spectrum disorders [50]. The ability of PEA to modulate neuroinflammation and associated symptoms (i.e., pain, depression, and anxiety) makes it a valid therapeutic tool in the treatment of several disorders. 

The goal of our study was to investigate the activity of PEA on two PTX-induced side effects, i.e., mood disorders and peripheral neuropathy (CIPN), and to understand whether PPAR-α and the endocannabinoid system play a role. Moreover, we evaluated central and peripheral inflammatory modulation processes using this bioactive lipid. For our study, the ultramicronized formulation (um-PEA) was used, due to its higher solubility and bioavailability after systemic administration [51].

## 2. Materials and Methods

### 2.1. Animals

CD1 male mice (3 months old, 25–30 g, Charles Rivers, Calco, Lecco, Italy) were placed in a controlled area (room maintained at 22 ± 1 °C with 12 h light/dark cycle) and supplied with water and food ad libitum, in the animal care facility at the Department of Pharmacy of the University of Naples Federico II, Italy. 

### 2.2. Drug Treatment 

Paclitaxel (PTX, Cat#S1150 Selleckchem, Houston, TX, USA) at the dose of 8 mg/kg was dissolved in a solution made up of 5% DMSO, 40% PEG 300, 5% Tween 80, and ddH_2_O according to the manufacture’s guidelines. Ultramicronized palmitoylethanolamide (um-PEA), kindly provided by Epitech Group SpA (Saccolongo, Italy), was dissolved in 1% carboxymethylcellulose. The CB1 antagonist, AM281, and the PPAR- α antagonist GW6471, obtained from Tocris Cookson (Bristol, UK), were dissolved in 4% DMSO and saline. 

### 2.3. Experimental Groups and Procedures

Mice were divided into five groups of *n* = 8 mice each, as follows:

- **Vehicle:** mice receiving saline intraperitoneally (IP).
- **PTX:** mice receiving PTX (8 mg/kg, 100 µL/mouse) IP at day 1, 3, 5, and 7.
- **PTX + um - PEA:** mice receiving PTX and then um-PEA (30 mg/kg for 7 days) by oral gavage.
- **PTX + um - PEA+AM281**: mice receiving PTX, then um-PEA, and on the last day AM281 (1 mg/kg) IP.
- **PTX+um-PEA+GW6471:** mice receiving PTX, then um-PEA, and on the last day GW6471 (2 mg/kg) IP.

Briefly, animals received PTX or vehicle treatments every other day for a week (days 1, 3, 5, and 7), as described previously [42,52]. One hour after the last day of PTX injection, mice started to receive oral um-PEA administration for 7 days. For antagonism experiments, on the last day of um-PEA injection, a different set of mice received AM281 or GW6471 IP 1 h before the um-PEA injection. The doses of AM281 and GW6471 selected in this study were based on the results of published data [41]. On the last day, mice were subjected to behavioral tests 1 h after um-PEA administration, and after euthanasia, the hippocampus and spinal cord were collected for ex vivo analysis. 

### 2.4. Behavioral Tests

#### 2.4.1. Depressive-like Behavior

Tail suspension test (TST). Mice were individually suspended by the tail 30–40 cm above the floor, using adhesive tape. The duration of immobility, recorded in seconds using a timer, was recorded during the 6-min test. Mice were considered immobile when they did not show any body movement, hung passively, with the absence of escape-oriented behavior.

Forced swimming test (FST). Mice were gently placed into individual glass cylinders (30 cm × 45 cm) filled with water maintained at 27 °C for 10 min, and their immobility times were recorded using a timer. Mice were considered immobile when floating in an upright position and only making small movements to keep their head above water, but without displacement. After the test, mice were allowed to dry and return to their home cage.

#### 2.4.2. Anxiety-like Behavior

Elevated plus-maze (EPM). The maze was composed of a central square, two open arms and two closed arms enclosed by vertical walls, placed 50 cm above the floor. Mice were individually placed on the central area of one of the open arms and allowed to move freely. The number of entries into the open arms during a 5-min exploration period were recorded and analyzed by video tracking software (Any-maze, Stoelting, Wood Dale, IL, USA). An entry was counted only if all four paws were inside the arm. At the end of the test, the apparatus was wiped with 70% ethanol.

Open field (OF) test. Mice were placed in an OF arena (25 cm × 25 cm), and locomotor activity (total distance travelled in meters) was recorded for 30 min and analyzed by video tracking software (Any-maze, Stoelting, Wood Dale, IL, USA). The apparatus was cleaned before and after each behavioral session with a solution of 70% ethanol.

#### 2.4.3. Pain Behavior 

Mechanical allodynia (von Frey test). To assess changes in sensation or in the development of mechanical allodynia, sensitivity to tactile stimulation was measured using a dynamic plantar aesthesiometer (DPA, Ugo Basile, Italy). Animals were placed in a chamber with a mesh metal floor covered by a plastic dome that enabled the animals to walk freely, but not to jump. The mechanical stimulus (paw withdrawal threshold) was then delivered to the mid-plantar skin of the hind paw. The cutoff was fixed at 5 g, while the increasing force rate (ramp duration) was settled at 20 s. The DPA automatically records the force at which the foot is withdrawn and the withdrawal latency. Each paw was tested twice per session. This test did not require any special pretraining, just an acclimation period to the environment and testing procedure.

Mechanical hyperalgesia (Randall–Selitto test). Mechanical hyperalgesia was assessed using a Randall–Selitto algesimeter (Ugo Basile). Before the test, each animal received 5 min of handling to get used to manipulation; then it was placed into a soft cotton cloth and carefully immobilized with the same hand used to hold the tested paw. The test consisted of the application of an increasing mechanical force, in which the tip of the device was applied onto the medial portion of the plantar surfaces until a withdrawal response resulted. The maximum force applied was limited to 200 g to avoid skin damage. 

Thermal allodynia (cold test). Cold sensitivity was measured as the number of foot withdrawal responses after application of acetone to the dorsal surface of the paw. A drop of acetone was applied to the dorsal surface of the paw with a syringe connected to a thin polyethylene tube while the mice were standing on a metal mesh. A brisk foot withdrawal response after the spread of acetone over the dorsal surface of the paw was considered as a sign of cold allodynia (n° paw withdrawal). 

Thermal hyperalgesia (plantar test). Heat hypersensitivity was assessed using the mice plantar test apparatus (Ugo Basile, Italy). The plantar test consisted of three Perspex boxes (22 × 19 × 25 cm) on an elevated glass table. Mice were housed in each box and left to acclimatize for at least 10 min. A mobile infrared heat source was applied to the plantar surface of the hind paws. The paw withdrawal latency was defined as the time (expressed in seconds) taken by the mice to remove its hind paw from the heat source. The heat source was calibrated to 15 IR intensity, and a cutoff point of 60 s was applied to prevent tissue damage. 

### 2.5. Ex Vivo Experiments

#### 2.5.1. Determination of Brain and Spinal Cord Markers of Inflammation 

The hippocampus and spinal cord samples were collected and TNF-α, IL-1β, IL-6, and IL-10, COX-2, and iNOS levels were measured using real time (RT)-PCR. For RT-PCR, total RNA was extracted from brain areas using TRIzol Reagent (Bio-Rad Laboratories, Hercules, California, USA) according to the manufacturer’s instructions. cDNA from 4 µg total RNA was retrotranscribed using a reverse transcription kit (NucleoSpin^®^, MACHEREY-NAGEL GmbH & Co, Düren, Germany). RT-PCR reactions were performed using Bio-Rad CFX96 PCR System and relative software (Bio-Rad Laboratories). Mouse primers for TNF-α, IL-1β, IL-6, IL-10, COX-2, and iNOS were purchased from Qiagen (Hilden, Germany). Glyceraldehyde 3-phosphate dehydrogenase (GAPDH) was used as a housekeeping gene for normalization. Data are expressed using the ΔΔCT method.

#### 2.5.2. Western Blotting 

Spinal cord samples were homogenized on ice-cold lysis buffer (20 mM Tris–HCl (pH 7.5), 10 mM NaF, 150 mM NaCl, 1% Nonidet P-40, 1 mM phenylmethylsulfonyl fluoride, 1 mM Na3VO4, leupeptin and trypsin inhibitor 10 μg/mL; 0.25/50 mg tissue). After 1 h, tissue lysates were obtained by centrifugation at 2.0 × 10^4^ g for 15 min at 4 °C. Protein concentrations were estimated with the Bio-Rad protein assay (Bio-Rad Laboratories, Milan, Italy) using bovine serum albumin as standard. Lysate proteins were dissolved in Laemmli sample buffer, boiled for 5 min, and separated by SDS-polyacrylamide gel electrophoresis and transferred to a nitrocellulose membrane (240 mA for 40 min at room temperature). The filter was then blocked with 1×phosphate buffer saline (PBS) and 3% non-fat dried milk for 40 min at room temperature and probed with anti-peroxisome proliferator-activated receptor (PPAR)-α (dilution 1:1000, cat. no. P0369, Sigma-Aldrich, Milan, Italy), or anti-cannabinoid (CB) receptor 1 (dilution 1:1000, cat. no. NB120-23703, Novus Biologicals, Cambridge, UK) in 1×PBS, 3% non-fat dried milk, and 0.1% Tween 20 at 4 °C overnight. The secondary antibodies were incubated for 1 h at room temperature. Subsequently, the blots were extensively washed with PBS, developed using enhanced chemiluminescence detection reagents (Amersham Pharmacia Biotech, Piscataway, NJ, USA) according to the manufacturer’s instructions. The immune complex was visualized by the ChemiDoc Imaging System (Bio-Rad Laboratories). These blots were loaded with equal amounts of protein lysates, they were also incubated in the presence of the antibody against β-actin (cat. no. A5441, Sigma-Aldrich).

### 2.6. Statistical Analysis 

Statistical analyses were performed using Prism 9 GraphPad software (GraphPad Software Inc., San Diego, CA, USA). All in vivo data are presented as mean ± SEM. For all experimental data, the significances of the differences between groups were determined by one-way repeated measures ANOVA, followed by post hoc Bonferroni’s multiple comparison test. A value of *p* < 0.05 was considered statistically significant for all tests.

## 3. Results

### 3.1. Effect of um-PEA on PTX-Induced Depressive- and Anxiety-like Behaviors

**Figure 1:**
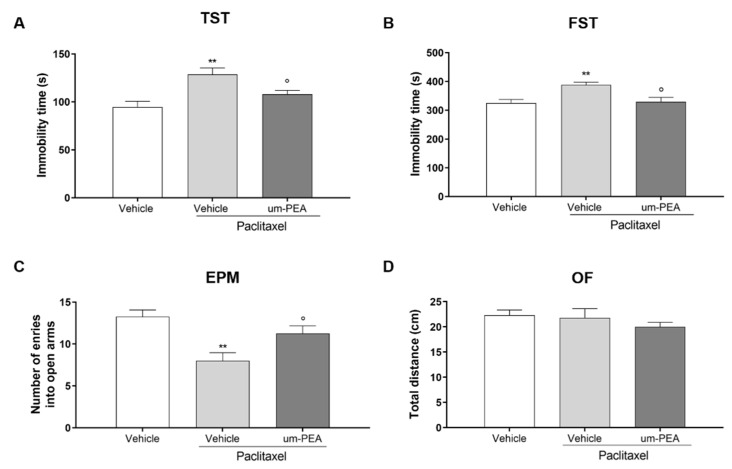
Effect of um-PEA on depressive- and anxiety-like behavior. (**A**) Time (in seconds) spent immobile in the TST; (**B**) time (in seconds) spent immobile in the FST test; (**C**) number of entries in the open arms of EPM test; (**D**) distance travelled (in cm) in the OF test; ** *p* < 0.01 versus vehicle group; ° *p* < 0.05 versus PTX group. Data are presented as mean ± SEM (*n* = 8). Differences were evaluated by ANOVA followed by Bonferroni’s post hoc test for multiple comparisons.

We investigated the possible effect of um-PEA in reducing PTX-induced depressive-like behaviors as assessed in the TST and FST. As expected, both tests showed that PTX increased immobility time compared to vehicle-treated mice (** *p* < 0.01; Figure 1A,B). Um-PEA administration for 7 days after the last PTX injection significantly reduced the time of immobility compared to vehicle-treated mice (° *p* < 0.05; Figure 1A,B).

The anxiety-like behavior was assessed in the EPM test. PTX-treated mice showed a significant decrease in the time spent in the open arms of the apparatus (** *p* < 0.001; Figure 1C) compared to vehicle-treated mice, which was significantly counteracted by repeated oral administration of um-PEA (° *p* < 0.05: Figure 1C). On the contrary, neither PTX nor- um-PEA had any effect on motor activity in the OF test (Figure 1D).

### 3.2. CB1 and PPAR-α Are Involved in um-PEA Central Activity

**Figure 2:**
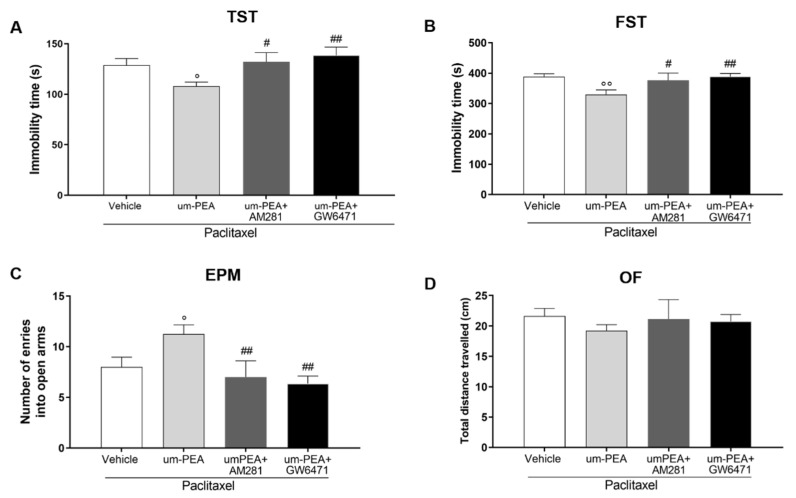
Effect of um-PEA on depressive- and anxiety-like behavior in presence of CB1 or PPAR-α antagonists. (**A**) Time (in seconds) spent immobile in the tail suspension test; (**B**) time (in seconds) spent immobile in the forced swim test; (**C**) number of entries in the open arms of elevated plus-maze test; (**D**) distance travelled (in cm) in the open field test; ° *p* < 0.05 and °° *p* < 0.01 versus PTX group; ^#^ *p* < 0.05 and ^##^ *p* < 0.01 versus PTX+ um-PEA. Data are presented as mean ± SEM (*n* = 8). Differences were evaluated by ANOVA followed by Bonferroni’s post hoc test for multiple comparisons.

Since it was reported that um-PEA acts as both a CB1 and a PPAR-α receptor agonist [24], and these receptors are localized in the brain [53,54], we further characterized um-PEA anxiolytic and antidepressant effects by performing behavioral tests in the presence of CB1 and PPAR-α antagonists, AM281 and GW6471, respectively. Both of the receptor antagonists significantly inhibited the antidepressant effect of um-PEA (^#^ *p* < 0.05, ^##^ *p* < 0.01; Figure 2A–B). Similar results were observed for the um-PEA anxiolytic effect compared to um-PEA+PTX-treated mice (^#^ *p* < 0.05, ^##^ *p* < 0.01; Figure 2C). Additionally, in this case, neither AM281 nor GW6471 changed the motor activity of the mice (Figure 2D).

### 3.3. Effect of um-PEA on Spinal and Sovraspinal Inflammatory Mediators in PTX Mice 

**Figure 3:**
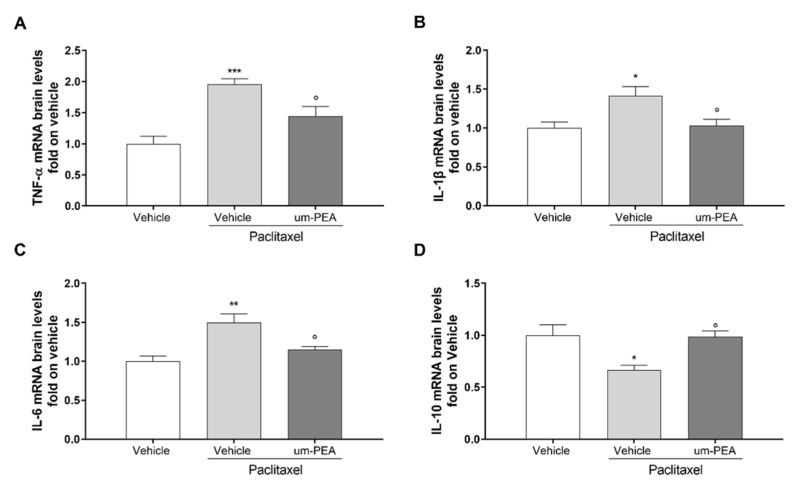
Pro-inflammatory cytokine levels in the hippocampi of vehicle- or um-PEA-treated mice injected with PTX. (**A**) Fold expression of mRNA for pro-inflammatory TNF-α; (**B**) fold expression of mRNA for pro-inflammatory IL-1β; (**C**) fold expression of mRNA for pro-inflammatory IL-6; (**D**) fold expression of mRNA for anti-inflammatory IL-10; * *p* < 0.05, ** *p* < 0.01, *** *p* < 0.001 versus vehicle group; ° *p* < 0.05 versus PTX group. Data are presented as mean ± SEM (*n* = 8). Differences were evaluated by ANOVA followed by Bonferroni’s post hoc test for multiple comparisons.

**Figure 4:**
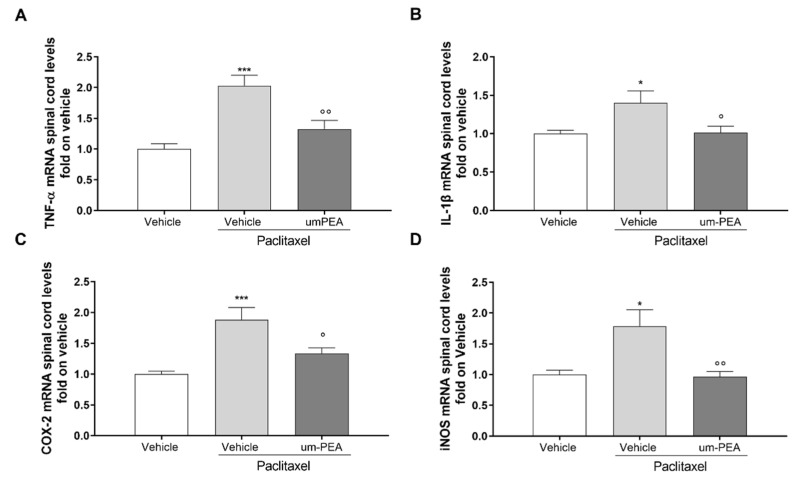
Pro-inflammatory cytokine levels in the spinal cords of vehicle- or um-PEA-treated mice injected with PTX. (**A**) Fold expression of mRNA for pro-inflammatory TNF-α; (**B**) fold expression of mRNA for pro-inflammatory IL-1β; (**C**) fold expression of mRNA for pro-inflammatory COX-2; (**D**) fold expression of mRNA for anti-inflammatory iNOS; * *p* < 0.05 and *** *p* < 0.001 versus vehicle group; ° *p* < 0.05 and °° *p* < 0.01 versus PTX group. Results are shown as mean ± SEM. Differences were analyzed using ANOVA followed by Bonferroni’s post hoc test for multiple comparisons.

The mRNA levels of pro-inflammatory cytokines in hippocampus and spinal cord tissues were analyzed using RT-PCR. A significant (* *p* < 0.05, ** *p* < 0.01, and *** *p* < 0.001 versus vehicle) induction in the expression of these cytokines was observed in the PTX-treated group at both the central (Figure 3A–D) and the spinal level (Figure 4A,B). Um-PEA treatment significantly decreased all of the pro-inflammatory cytokine gene levels analyzed (TNF-α, IL-1β, and IL-6), while increasing the levels of IL-10 (* *p* < 0.05 versus PTX; Figure 3A–D). In the spinal cord, um-PEA treatment significantly decreased pro-inflammatory cytokine gene levels of TNF-α and IL-1β (° *p* < 0.05 and °° *p* < 0.01 versus PTX; Figure 4A-B). To further determine the inhibitory effect of um-PEA on inflammatory mediators in the spinal cord, we also evaluated the mRNA expression level of iNOS and COX-2. There was an upregulation of iNOS and COX-2 mRNA expression after PTX treatment, which was significantly (* *p* < 0.05 and *** *p* < 0.001 versus vehicle) reduced by um-PEA treatment (° *p* < 0.05 and °° *p* < 0.01 versus PTX; Figure 4C, D).

### 3.4. Effect of um-PEA on PTX-Induced Peripheral Neuropathy 

**Figure 5:**
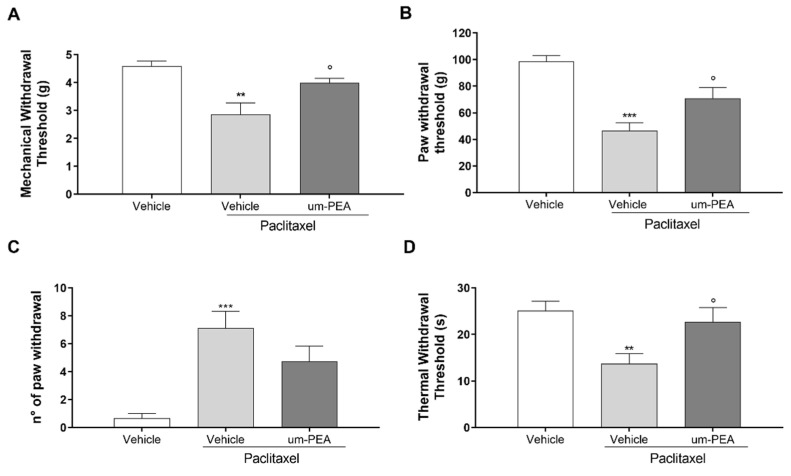
Effect of um-PEA on peripheral neuropathy. Results of the (**A**) von Frey test to evaluate mechanical allodynia; (**B**) Randall–Selitto test to assess mechanical hyperalgesia; (**C**) acetone evaporation test to evaluate thermal allodynia; (**D**) Hargreaves test to evaluate thermal hyperalgesia; ** *p* < 0.01; *** *p* < 0.001 versus vehicle group; ° *p* < 0.05 versus PTX group. Data are presented as mean ± SEM (*n* = 8). Differences were evaluated by ANOVA followed by Bonferroni’s post hoc test for multiple comparisons.

PTX treatment induced neuropathic pain; indeed, PTX-treated mice showed a significant reduction in mechanical allodynia (** *p* < 0.01) and hyperalgesia (*** *p* < 0.001) compared to the vehicle group (Figure 5A–B). Um-PEA treatment significantly counteracted the effect of PTX in both tests (° *p* < 0.05; Figure 5A-B). PTX treatment also induced marked cold allodynia and thermal hyperalgesia, resulting in a significant increase in cold responses (*** *p* < 0.001) and significant decrease in thermal nociceptive thresholds (** *p* < 0.01) compared to vehicle group (Figure 5C,D). In contrast, um-PEA administration produced a significant reduction in the number of paw withdrawals in the acetone test, and enhanced the thermal withdrawal thresholds in the Hargreaves test (° *p* < 0.05) compared to PTX animals (Figure 5C,D).

### 3.5. Effect of um-PEA in PTX-Treated Mice Is PPAR-α and CB1 Mediated

**Figure 6:**
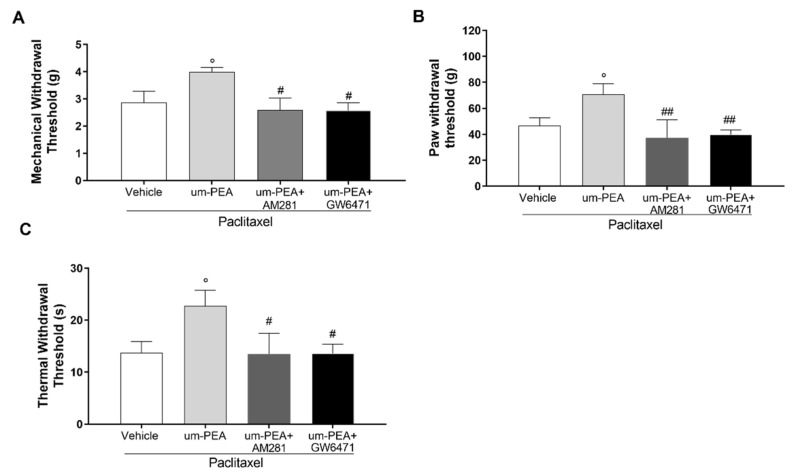
Effect of um-PEA on peripheral neuropathy in presence of CB1 or PPAR-α antagonists. Results of the (**A**) von Frey test to evaluate mechanical allodynia; (**B**) Randall–Selitto test to assess mechanical hyperalgesia; (**C**) Hargreaves test to evaluate thermal hyperalgesia; ^#^ *p* < 0.05 and ^##^ *p* < 0.01 versus PTX+um-PEA; ° *p* < 0.05 versus PTX-vehicle group. Data are presented as mean ± SEM (*n* = 8). Differences were evaluated by ANOVA followed by Bonferroni’s post hoc test for multiple comparisons.

The antinociceptive action of um-PEA was investigated in the presence of a selective CB1 receptor antagonist (AM281) and a selective PPAR-α antagonist (GW6471). As expected, the increase in paw withdrawal threshold of um-PEA-treated mice was reversed to a significant extent by AM281 in the Randall–Selitto, von Frey, and Hargreaves tests, compared to um-PEA-treated mice (^##^ *p* < 0.01, Figure 6A–C). Additionally, we found that the analgesic effect of um-PEA was also significantly counteracted in mice treated with the selective PPAR-α antagonist (GW6471). Indeed, GW6471 administration in the um-PEA-treated group resulted in significant hyperalgesic and allodynic effects (^##^ *p* < 0.01, Figure 6A–C). 

**Figure 7:**
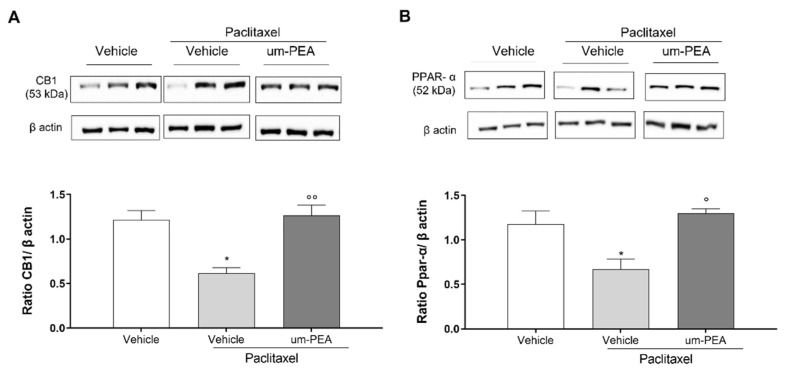
Western blotting analyses for CB1 (**A**) and PPAR-α (**B**) receptors in spinal cords. Immunoblots representative of all tissues analyzed are shown. Densitometric analysis of protein bands are reported: the levels are expressed as the density ratio of target to control protein bands (β-actin). * *p* < 0.05 versus vehicle group; ° *p* < 0.05 and °° *p* < 0.005 versus PTX group. Values are expressed as mean ± SEM (*n* = 3). Differences were evaluated by ANOVA followed by Bonferroni’s post hoc test for multiple comparisons.

Finally, in order to evaluate the roles of CB1 and PPAR-α receptors in PTX-induced neuropathy, ex vivo experiments were conducted. By Western blot analysis, we confirmed that PTX was involved in the maintenance of pain hypersensitivity by mechanical stimuli, since it was able to reduce CB1 receptor expression in the spinal cord compared to the vehicle group (Figure 7A–B, * *p* < 0.05). Um-PEA administration increased significantly CB1 receptor expression (Figure 7A, ^°°^ *p* < 0.01). No significant differences between vehicle and PTX+um-PEA groups were observed. Moreover, we also evaluated PPAR-α expression in the spinal cord, since this receptor has an important and well-known role in inflammation control. PTX reduced its expression (Figure 7B, * *p* < 0.05), while um-PEA was able to restore it by a significant degree (^#^ *p* < 0.05).

## 4. Discussion

The use of PTX as a chemotherapeutic agent has become a broadly accepted option in the treatment of patients with ovarian, breast, non-small-cell lung cancers, malignant brain tumors, and a variety of other solid tumors [1]. Significant toxicities in both the brain and periphery limit the effectiveness of PTX-based treatment regimens; this is a crucial limiting factor that can lead to a change or reduction in therapy or, in severe cases, to its total cessation [1]. In this study, we evaluated the efficacy of um-PEA in reducing PTX-related side effects, i.e., peripheral neuropathy and mood alteration, and we also investigated the possible mechanisms of action of um-PEA. 

It is known that after the first cycle of PTX, it is already possible to observe the development of neuropathic pain and emotional disorders [55]. Accordingly, here we show that PTX-treated mice manifested anxiety-like behaviors in the EPM and OF tests, and depressive-like behaviors in the FST and TST. The possible mechanisms underlying these behavioral symptoms can be attributed to the development of neuroinflammation through glial cell activation [52] and/or the induction of central neurotoxicity. It is also possible that PTX sensitized the immune responses. Indeed, hypersensitivity to stimuli, not only in neuropathic pain, but also in inflammatory pain, can be explained by both peripheral and central sensitization of sensory nerve fibers [56]. 

It has been reported that during chronic inflammation, tissue levels of endogenous PEA are decreased, either due to reduced production or increased degradation [57] (or both). We thus hypothesized that treatment with a bioavailable form of PEA (i.e., um-PEA) could prevent or treat PTX-induced side effects. Our data show that 7 days of oral administration of um-PEA inhibited affective disorders in PTX mice. In detail, um-PEA reduced depressive- and anxiety-like behaviors, as shown in the TST, FST, and EPM test, respectively, while the OF test was not sensitive to PTX-induced changes. This result suggests that locomotion alterations did not influence the behavioral results. Several studies have indicated that PPAR signaling is involved in the regulation of anxiety responses. Indeed, Domi et al. [58] demonstrated that PPAR-γ antagonism induces an anxiogenic effect in mice, as detected both in the OF and EPM tests. Regarding the depressive-like behaviors associated with PTX administration, um-PEA treatment normalized the increase in the immobility time in the TST and FST. Several studies have already reported the potential antidepressant effects of PEA (either alone or in combination with antidepressants) [59,60], even in depressive-like behavior associated with neuropathic pain or traumatic injury [61]. Moreover, pharmacological inhibition of PEA degradation, as well as the upregulation of its biosynthesis, also resulted in antidepressant effects [62,63].

Although several molecular mechanisms have been suggested to explain PEA effects, its activity is mainly mediated by PPAR-α [59,64,65]. The activation of PPAR-α receptor initiates a cascade of events that causes the suppression of pain and inflammation, including decreases in pro-inflammatory cytokines such as IL-1β and IL-6, and TNF-α [57]. Moreover, low levels of PPAR-α are also responsible of several pathological conditions, neurodegenerative diseases, and stress-related disorders [66].

Here we found decreased levels of PPAR-α in the spinal cord of PTX mice, which was normalized by um-PEA. Although PPARs are not canonical endocannabinoid receptors, they are activated by several endocannabinoid mediators, and thus are considered as part of the enlarged endocannabinoid system, currently referred to as the endocannabinoidome [67]. Interestingly, evidence has shown that PEA, either by reducing anandamide (AEA) metabolism or binding to PPAR-α, upregulated the expression of CB receptors and increased TRPV1 activation, suggesting that PEA is able to interact both with the endocannabinoid and endovanilloid systems [68,69]. The CB1 receptor, which is highly expressed in the CNS, plays an important role in the regulation of stress and emotions [70,71]. Indeed, several studies have shown that CB1 agonists reduce neuroinflammation and have anxiolytic as well as antidepressant effects [72,73]. 

Therefore, one of the objectives of the study was to evaluate the protein expression of two main receptors involved in the beneficial effects of PEA, PPAR-α, and CB1 receptors. For instance, PEA exhibits analgesic effects via two different and distinct pathways, direct activation of PPAR-α or indirect activation of CB1 receptors, both of which relieve pain in different ways. Our results show that um-PEA counteracts the PTX-induced decrease in the expression not only of PPAR-α, but also CB1. Moreover, here we found that the effects of um-PEA were significantly inhibited by the administration of either AM281 (CB1 antagonist) or GW6471 (PPAR-α antagonist). As already mentioned, the reduction in or the absence of CB1 and PPAR-α could lead to neuroinflammation [66,74,75,76,77].

It is well documented that chronic administration of PEA is able to significantly reduce neuroinflammation [23,34,78], protect neurons from death [79,80], reduce oxygen radicals, and improve behavioral, motor, and cognitive deficits [81,82]. Neuroinflammation is a localized inflammation occurring in the PNS and CNS in response to trauma, bacterial/viral infection, autoimmunity, and/or toxins [83,84]. In particular, neuroinflammation is a common feature across different conditions, including neurodegenerative diseases, fibromyalgia, and chronic pain [83,85,86]. Different studies have reported the effect of PEA in the management of pain and inflammatory conditions [87,88,89]. The relationship between inflammation and pain is bidirectional, since the activation of pain circuits can also regulate neuroinflammation in the CNS [84]. Although acute neuroinflammation plays a protective role [90], its chronicization (i.e., non-resolving neuroinflammation) is detrimental, since the over-release of pro-inflammatory factors and cytokines can alter brain structure and function [91,92]. Non-resolving neuroinflammation is a key factor in the pathogenesis of CIPN, as shown by the significant increase in plasma levels of pro-inflammatory cytokines and chemokines involved in hypersensitivity and pain (e.g., IL-1β and TNF-α) in PTX-treated mice [93].

The decreased hippocampal gene expression of pro-inflammatory cytokines, which was here observed following 7-day oral administration of um-PEA, clearly shows the protective role of PEA-um against PTX-induced neuroinflammation. This central protective effect was also observed at the spinal cord level, since um-PEA treatment significantly decreased COX-2, iNOS, TNF-α, and IL-1β compared to the vehicle group. 

Based on these findings, we also evaluated the efficacy um-PEA in PTX-induced peripheral neuropathy. In fact, Donvito et al. [41] had already found that PEA reversed PTX-induced neuropathy in a dose dependent manner. In their study, a single administration of PEA was able to reverse mechanical allodynia through a PPAR-α-mediated mechanism. In our study, mice receiving um-PEA treatment beginning at the last PTX injection show a reduction not only in allodynia signs but also hyperalgesia. In agreement with our data, Di Cesare Mannelli and coworkers reported analgesic proprieties of um-PEA in oxaliplatin-induced neuropathy, and showed that this acylethanolamine prevented the development of mechanical hypersensitivity, with a significant anti-inflammatory effect also being observed [40]. Recently, we have confirmed that um-PEA exerts its analgesic and anti-inflammatory effects primarily through direct activation of the transcription factor PPAR-α [50]. In particular, PEA has been found to switch off the nuclear factor kB signaling pathway, a crucial element in the transcription of genes, leading to the synthesis of pro-inflammatory and pro-analgesic mediators [64,94]. 

Moreover, in a chronic constriction injury model of neuropathic pain, PEA not only reduced edema and macrophage infiltration, but also counteracted the decrease in axon diameter and myelin thickness, the effects being lost in PPAR-α-null mice [95]. Recently, it has also been reported that the activation of PPARs may interfere with the production of pro-inflammatory cytokines in CIPN, potentially attenuating and preventing the symptoms of neuropathy [96]. In agreement with all these studies, we here demonstrate that um-PEA reduces pro-inflammatory cytokines by PPAR-α activation. In fact, repeated um-PEA administration increased the spinal cord expression of the nuclear receptor, and the PPAR-α antagonist GW6471 reversed um-PEA analgesic effects. 

In addition, it is also interesting to note that the restoration of CB1 receptor levels in the spinal cord, following um-PEA treatment, seem to be important for pain control and inflammation reduction. We have also confirmed the correlation between PEA and the endocannabinoid system via both in vivo and ex vivo experiments, using AM281, a CB1 antagonist able to reverse um-PEA analgesic effects; moreover, repeated um-PEA administration preserves CB1 receptor expression in the spinal cord. Accordingly, a recent study reported that the administration of an analog of PEA, N-(4-methoxy-2-nitrophenyl)hexadecanamide (HD), produced a dose-dependent antinociceptive effect in rats, which was significantly counteracted by AM281 administration [97]. Taken together, these findings provide an overview of the crosstalk between PPARs and cannabinoids, and the importance of their reciprocal regulation in the control of major physiological and pathophysiological functions.

## 5. Conclusions

In summary, our results demonstrate that 7-day oral administration of um-PEA significantly reduced PTX side effects. Due to its anti-inflammatory activity and marked analgesic proprieties, as well as its ability to activate PPAR-α and influence the endocannabinoid system, um-PEA is a good candidate for the management of neuropathic pain and mood disorders produced by chemotherapy.

## Data Availability

Not applicable.
